# SnO_2_–Fe_3_O_4_ nanocomposites for the photodegradation of the Congo red dye

**DOI:** 10.1016/j.heliyon.2022.e09204

**Published:** 2022-03-31

**Authors:** Muhammad Said, Widya Twiny Rizki, Wan Ryan Asri, Desnelli Desnelli, Addy Rachmat, Poedji Loekitowati Hariani

**Affiliations:** a Department of Chemistry, Faculty of Mathematics and Natural Sciences, Sriwijaya University, Jalan Palembang-Prabumulih Km 32 Indralaya, Sumatra Selatan, Indonesia; b Research Centre of Advanced Material and Nanocomposite, Faculty of Mathematics and Natural Science, Sriwijaya University, Jalan Palembang Prabumulih Km 32 Indralaya, Sumatera Selatan, Indonesia

**Keywords:** SnO_2_–Fe_3_O_4_, Nanocomposite, Photodegradation, Congo red, Hydrothermal

## Abstract

Synthesis of SnO_2_–Fe_3_O_4_ nanocomposites was conducted. The purpose of this study was to obtain the SnO_2_–Fe_3_O_4_ nanocomposites, the effectiveness of photodegradation Congo red by SnO_2_–Fe_3_O_4_ nanocomposites and determine the kinetics of photodegradation. The XRD analysis showed that the SnO_2_–Fe_3_O_4_ nanocomposite best mass ratio was 1:1 based on the highest intensity of characteristic angle (2θ), which is 26.54° and had the smallest crystal size, which is 9.662 nm. Based on the UV-Vis DRS result, the SnO_2_–Fe_3_O_4_ nanocomposites bandgap value was 2.3 eV. The magnetization saturation value of SnO_2_–Fe_3_O_4_ nanocomposites was 64.96 emu/g. The morphology of SnO_2_–Fe_3_O_4_ nanocomposites showed by the TEM image, where the dark spots spread in the lighter areas. The surface of SnO_2_–Fe_3_O_4_ nanocomposites characterized by SEM with the result was bumpy surface and many pores. The EDS result of SnO_2_–Fe_3_O_4_ nanocomposites confirmed the presence of Fe, Sn, and O elements. The functional group of SnO_2_–Fe_3_O_4_ nanocomposites showed by FTIR data, the stretch band of Sn–O characteristics showed at wavenumber 590 cm^−1^, and the stretch band of Fe–O showed at wavenumber 563 cm^−1^. The optimum condition of nanocomposites at a contact time of 90 min and the optimum concentration of 18 mg/L showed that the percent of photodegradation was 50.76%. The photodegradation rate of SnO_2_–Fe_3_O_4_ was fitted to Pseudo-second-order.

## 1 Introduction

SnO_2_ was reported by Wu et al. [1] to possess a photocatalytic ability because it degrades dyes and other organic compounds. This photocatalytic ability is usually associated with semiconductors and it is improved through modification and the development of composites. Bharati et al. [2] stated that Fe_3_O_4,_ a nanomagnetic material is a semiconductor that provides the best effect in photodegradation and stability in water media. Gandhoor et al. [3] showed that Fe_3_O_4_ was classified as a semiconductor with a bandgap of 0.3 eV. Therefore, it was described to possess the strongest magnetic properties among other iron oxides.

Several methods are utilized in the synthesis of semiconductor oxide nanoparticles, namely sol-gel [4], flame spray [5], and hydrothermal [6]. The hydrothermal method is a process where water and heat are utilized to convert solutions into crystals. It is usually carried out in a closed system to prevent the loss of solvent when heated above its boiling point [7]. One of the advantages of the hydrothermal method is that it produces higher crystallinity and purity [8]. According to Wang et al. [9], which synthesized SnO_2_–Fe_3_O_4_ using the hydrothermal method, it also showed its importance. In this study, two methods of SnO_2_–Fe_3_O_4_ synthesis were combined, namely hydrothermal and coprecipitation. By combining the two processes, it was expected that the resulting nanocomposite possesses high purity and magnetic properties.

Recently, the industrialization process, technological advances, and the declining availability of clean water have become major concerns. Various contaminants such as toxic chemicals, phosphates, heavy metals, dyes are present, causing a decrease in water quality [10]. Among the pollutants that have been reported, dyes are the primary pollutant in wastewater and are often present in large quantities from various sources [11]. Specifically, cationic dye such as Congo red is hazardous because it is toxic, carcinogenic, and causes various problems. This dye is also difficult to naturally degrade in the environment [12]. Therefore, it is highly recommended to remove pollutants from wastewater before being discharged into water resources or the environment as well.

Several researchers have experimented with different dye degradation processes, including the use of microorganisms [13] and photocatalysts [14]. The photocatalyst method is a fairly efficient process compared to others, because it decomposes dye compounds into harmless chemicals such as H_2_O and CO_2_.

## 2 Materials and methods

### 2.1 Materials

The materials used in this study were distilled water, hydrochloric acid (HCl) (37–38% purity), sulfuric acid (H_2_SO_4_) (95–97% purity), hydrogen peroxide (H_2_O_2_) (30% purity), ethanol absolute (≥99.9% purity), tin (II) chloride dihydrate (SnCl_2_.2H_2_O) (98.0–103.0% purity), iron (III) chloride hexahydrate (FeCl_3_.6H_2_O) (99.0–102.0%, purity), iron (II) chloride tetrahydrate (FeCl_2_.4H_2_O) (≥98.0 %, purity), sodium hydroxide (NaOH) (≥97.0 %, purity), sodium nitrate (NaNO_3_) (≥99.5 %, purity), sodium chloride (NaCl) (≥99.5 %, purity), dye congo red (C_32_H_22_N_6_Na_2_O_6_S_2_) (dye content ≥35 %). All chemicals were purchased from Merck, Germany.

### 2.2 Synthesis of SnO_2_

One gram of SnCl_2_.2H_2_O was dissolved in 50 mL of distilled water and 25 mL absolute ethanol, then it was stirred for 2 h. After that the solution was adjusted to pH 2 and was transferred to Teflon, then it was heated hydrothermally in an oven at 150 °C for 12 h [15].

### 2.3 Synthesis of Fe_3_O_4_

Fe_3_O_4_ was prepared by dissolving 1.99 g of FeCl_2_.4H_2_O and 5.41 g FeCl_3_.6H_2_O in 100 mL of aquademin. Next, 1M NaOH was gradually added to the mixture while stirring with a stirrer at 70 °C until a black precipitate was formed and the pH was ±12. Then the precipitate was dried using an oven at 70 °C for 3 h [16].

### 2.4 Synthesis of SnO_2_–Fe_3_O_4_ nanocomposites

SnO_2_–Fe_3_O_4_ nanocomposites were prepared using coprecipitation and hydrothermal methods. After the synthesis of Fe_3_O_4_, various mass ratios of FeCl_2_.4H_2_O and FeCl_3_.6H_2_O were produced as shown in Table 1. Then the Fe_3_O_4_ obtained was mixed with various amounts of SnCl_2_.2H_2_O as shown in Table 1. The solution was adjusted to pH 2 and was placed in Teflon then it was heated hydrothermally in an oven at 150 °C for 12 h. The precipitate obtained was dried at 70 °C for 24 h [9].

**Table 1:** Comparison of the mass of SnO_2_–Fe_3_O_4_.

Ratio SnO_2_–Fe_3_O_4_ Nanocomposites	SnCl_2_.2H_2_O (g)	FeCl_2_.4H_2_O (g)	FeCl_3_.6H_2_O (g)
1:1	1	1.99	5.41
1:2	1	3.98	10.82
2:1	2	1.99	5.41

The prepared nanocomposites were characterized using spectrophotometer FT-IR Thermo Scientific Nicolet iS10, Scanning Electron Microscope- Energy Dispersive X-ray Spectrometry (SEM-EDS) VEGA 3 TESCAN, and UV-Vis Orion Aquamate 8000. Also, UV-VIS Diffuse Reflectance Spectroscopy (UV-Vis DRS) UV-1700 Series, Transmission Electron Microscopy (TEM) JEOL JEM 1400, Vibrating Sample Magnetometer (VSM) Lakeshore 74004, X-Ray diffractometer (XRD) Rigaku Miniflex-600 were utilized.

### 2.5 Photodegradation study

Photodegradation batch studies were conducted in a UV light photoreactor with a wavenumber of 254 nm and a power of 14 W (Philips). Determination of the optimum conditions was conducted in sequence by each variable. 0.03 g of nanocomposite was mixed with 30 mL of Congo Red with initial dye concentrations variations of 6, 10, 14, 18 and 22 mg/L and H_2_O_2_ volume variations of 0.1, 0.2, 0.3, 0.4, 0.5, 0.75 mL. Subsequently, it was irradiated in a UV photoreactor with irradiation time variations for 30, 45, 60, 75, 90, and 105 min and stirred using a magnetic stirrer. After that, the solution was separated from the nanomagnets using a permanent magnet. The absorbance of the filtrate was measured at the maximum wavelength of Congo red (449 nm). The photodegradation using SnO_2_ and SnO_2_–Fe_3_O_4_ nanocomposite without UV lamp irradiation was also carried out as a control experiment with the same procedure.

## 3 Results and discussion

### 3.1 Characterization using XRD

**Figure 1:**
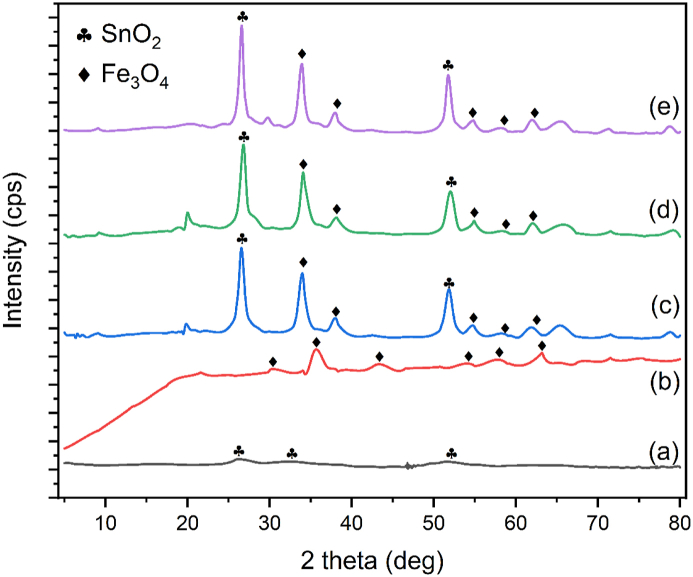
XRD spectrum of (a) SnO_2_ (b) Fe_3_O_4_ (c) SnO_2_–Fe_3_O_4_ (1:1) (d) SnO_2_–Fe_3_O_4_ (1:2) and (e) SnO_2_–Fe_3_O_4_ (2:1) nanocomposites.

Synthesis of SnO_2_ in this research was conducted according to the standard reaction. Then the resultant was compared with the standard-issue by Joint Committee on Powder Diffraction (JCPDS) No. 88–0287. Based on Figure 1 (a) which showed the diffraction pattern of SnO_2_ nanomagnetic X-rays, where the peak widened at the centre of 2θ at 26.52, 32.1, and 51.8. Data from the XRD characterization results and particle size calculations for SnO_2_ using the Debye-Scherer formula, showed that the resultant size of SnO_2_ was 2,829 nm. This indicated that it has a nanoparticle shape because it has a particle size that was below 100 nm.

The magnetic properties of the synthesized nanoparticles were characterized using XRD which showed a diffraction angle of 2 θ, peak intensity, and different types of phases. Furthermore, it was conducted by compa § ring the peaks formed on the Fe_3_O_4_ diffractogram with that of the JCPDS data. Based on the standard-issue by Joint Committee on Powder Diffraction (JCPDS) No. 65–30107, five peaks of 2θ characterizes this compound, namely 30.205 °, 35.515 °, 43.325 °, 53.711 °; 57.215 ° and 62.76 ° with field indexes (220), (311), (400), (422), (511), and (440). The diffraction Fe_3_O_4_ sample showed sharp peaks at an angle of 30.52°, 35.86°, 43.44°, 53.72°, 57.2° and 62.76°. This finding was consistent with previous reports [17].

Based on Figure 1 the diffraction pattern of Fe_3_O_4_ nanomagnetic X-rays in which the peak widened at the centre were 35.86 and 57.2, indicating the successful synthesis of magnetite. Then, data were obtained from XRD characterization and particle size calculations for magnetite nanoparticles using the Debye-Scherer formula. The resultant size of the magnetite nanoparticle was 13.66 nm, which showed that Fe_3_O_4_ was a nanoparticle because it has a particle size that was below 100 nm and the crystal size of 122.66 nm.

The synthesis of SnO_2_–Fe_3_O_4_ nanocomposite with various mass ratios (1:1), (1:2) and (2:1) was carried out using the hydrothermal method. Based on Figure 1, the 2θ angle in the SnO_2_–Fe_3_O_4_ (1:1), (1:2), and (2:1) composite had almost the same characteristics as that of SnO_2,_ especially at 26.52°. The peak intensity of the SnO_2_–Fe_3_O_4_ composite was observed to decrease with the addition of SnO_2_ in the composite. Based on Wang et al. [9], the composite in comparison with the intensity decreased with the SnO_2_ peak covered by the crystallite of magnetite nanoparticles. From the XRD results the best ratio of SnO_2_–Fe_3_O_4_ synthesis occurred at a mass ratio of 1:2, showing the highest intensity around the angle of 2θ = 26.74° and 34.08°. Based on the data from XRD characterization and particle size calculations for SnO_2_–Fe_3_O_4_ using the Debye-Scherer formula the obtained size of SnO_2_–Fe_3_O_4_ (1:1) was 9.833 nm. While that of SnO_2_–Fe_3_O_4_ (1:2) was 9.662 nm and SnO_2_–Fe_3_O_4_ (2:1) was 13.543 nm which showed that SnO_2_–Fe_3_O_4_ was a nanoparticle because it had a particle size that was below 100 nm.

### 3.2 Characterization using UV-VIS DRS

**Figure 2:**
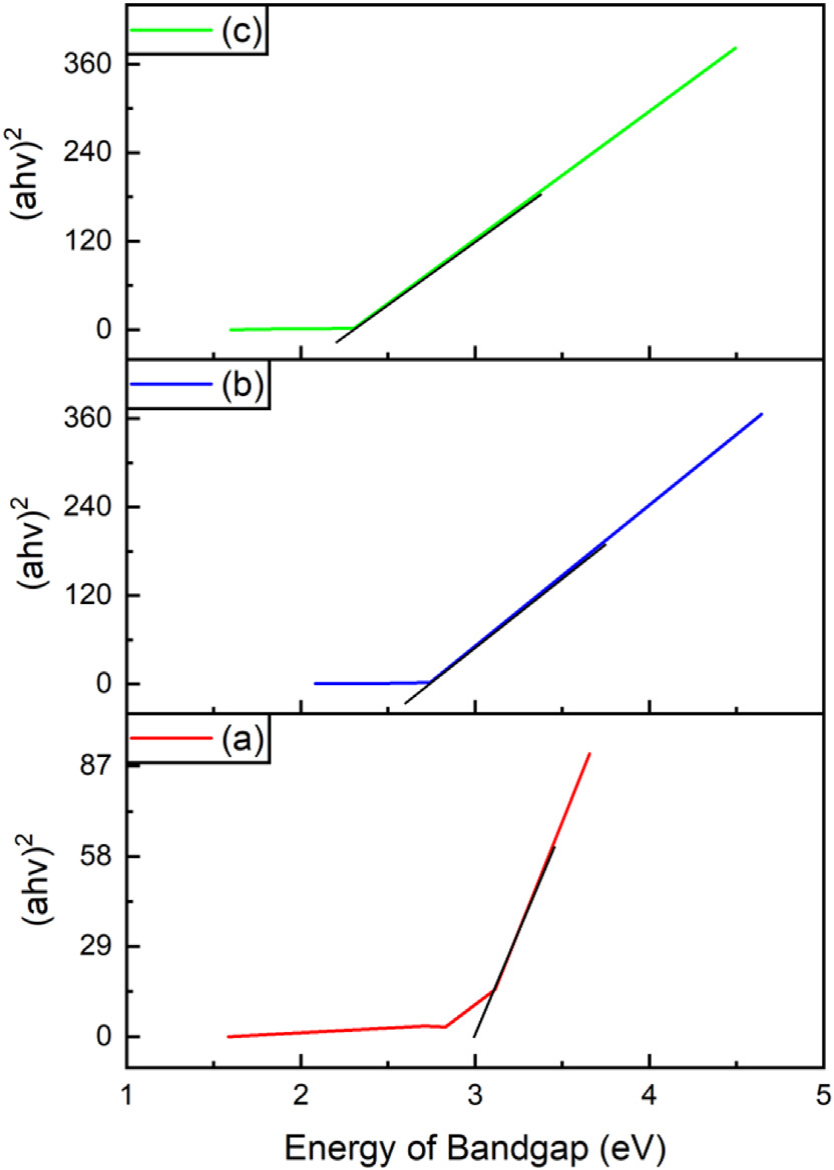
UV-Vis DRS (a) SnO_2_, (b) Fe_3_O_4_ and (c) SnO_2_–Fe_3_O_4_ nanocomposite.

SnO_2_, Fe_3_O_4_ and SnO_2_–Fe_3_O_4_ nanocomposites were analyzed using UV-Vis DRS. The purpose of this was to determine the value of the energy bandgap and to observe the changes that occurred in SnO_2_ when it was combined with Fe_3_O_4_. The energy band gap is the distance between the two bands in a semiconductor, i.e., the distance from the valence, to the conduction band. Characterization using UV-Vis DRS was carried out by providing energy at 200–800 nm. Causing excitation of electrons from the valence to the conduction band, then the electrons returned to their ground state by transmitting a certain amount of energy, which was proportional to that of the bandgap. The results of measuring the bandgap energy using the UV-Vis DRS are presented in Figure 2.

Based on Figure 2, the bandgap energy for SnO_2_ was 3.05 eV, and according to Huda et al. [15], SnO_2_ is a semiconductor with an energy band gap of 3.160 eV. This showed that the resulting energy band gap was smaller than the standard for SnO_2_, which is 3.6 eV. Furthermore, the synthesized Fe_3_O_4_ had bandgap energy of 2.7 eV, and that of the SnO_2_–Fe_3_O_4_ nanocomposite was 2.3 eV, therefore it was concluded that the SnO_2_–Fe_3_O_4_ nanocomposite was in the semiconductor range. The change in the bandgap energy in the nanocomposites was due to the effect of those in the synthesized Fe_3_O_4_ which was smaller than that of SnO_2_. The bandgap value of the SnO_2_–Fe_3_O_4_ nanocomposite which was smaller than SnO_2_ affected the photodegradation power. The smaller the bandgap size, the greater the photocatalytic properties. According to Wei et al. [18], a good photocatalyst is that which has a low bandgap, because the area with a wider wavelength causes UV absorption and results in more electrons and holes during the photocatalytic reaction process.

### 3.3 Characterization using VSM

**Figure 3:**
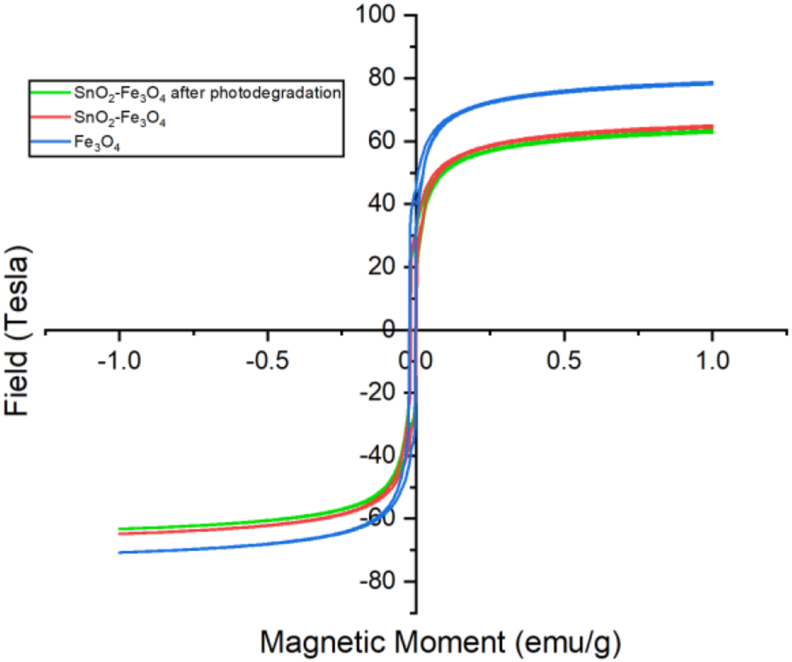
Hysteresis curve of Fe_3_O_4_, SnO_2_–Fe_3_O_4_ nanocomposites and SnO_2_–Fe_3_O_4_ nanocomposites after photodegradation.

This characterization was carried out using a VSM tool, and it was aimed to determine the magnitude of the magnetic properties due to changes in the external field of Fe_3_O_4_. And also to determine changes in the magnetic field of SnO_2_–Fe_3_O_4_ nanocomposites and the alterations after being applied as a photocatalyst. The information obtained was in the form of magnetic properties as described by the hysteresis curve, which stated the relationship between magnetization (M) and the external magnetic field (H). The hysteresis curve resulting from the VSM characterization is shown in Figure 3.

Based on Figure 3, Fe_3_O_4_ had the highest saturation magnetization value at 78.76 emu/g. These results changed with the nanomagnetic saturation moment of pure Fe_3_O_4_, which was 92 emu/g [19]. The saturation magnetization value was influenced by the presence of an impurity on the magnetic particles. There was a decrease in the saturation magnetization value of the SnO_2_–Fe_3_O_4_ nanocomposite which was smaller than that of Fe_3_O_4_. The decrease was caused by the addition of nonmagnetic SnO_2_ which coated Fe_3_O_4_. According to Kuppan et al. [20] SnO_2_ is a particle that has super nonmagnetic properties. The decrease in the saturation magnetization value was also influenced by the density of the particles which resulted in easy interaction that led to a magnetic moment. This is because the denser the particles, the greater the number of the magnetic moment. Based on the data, the VSM nanocomposite had a saturation value of 64.96 emu/g, which means that it had good superparamagnetic properties. Although SnO_2_–Fe_3_O_4_ nanocomposites had lower magnetic saturation values than Fe_3_O_4_, they still possessed the magnetic properties needed for separation.

SnO_2_–Fe_3_O_4_ nanocomposite was applied as a photocatalyst to degrade the Congo red dye. The role of Fe_3_O_4_ present in it was to provide magnetic properties in order to aid the separation of SnO_2_ from water, for it to be reused in the next photocatalytic process. After the photodegradation of the nanocomposites, the VSM measurement was again carried out to observe the changes in the magnetic saturation value. Which discovered that the magnetic saturation value of SnO_2_–Fe_3_O_4_ nanocomposite after photodegradation was not too different than before it, at 63.40 emu/g. This proves that the SnO_2_–Fe_3_O_4_ nanocomposite is environmentally friendly because it has the potential of being reused for subsequent photodegradation.

### 3.4 Characterization of SnO_2_–Fe_3_O_4_ nanocomposites using TEM

**Figure 4:**
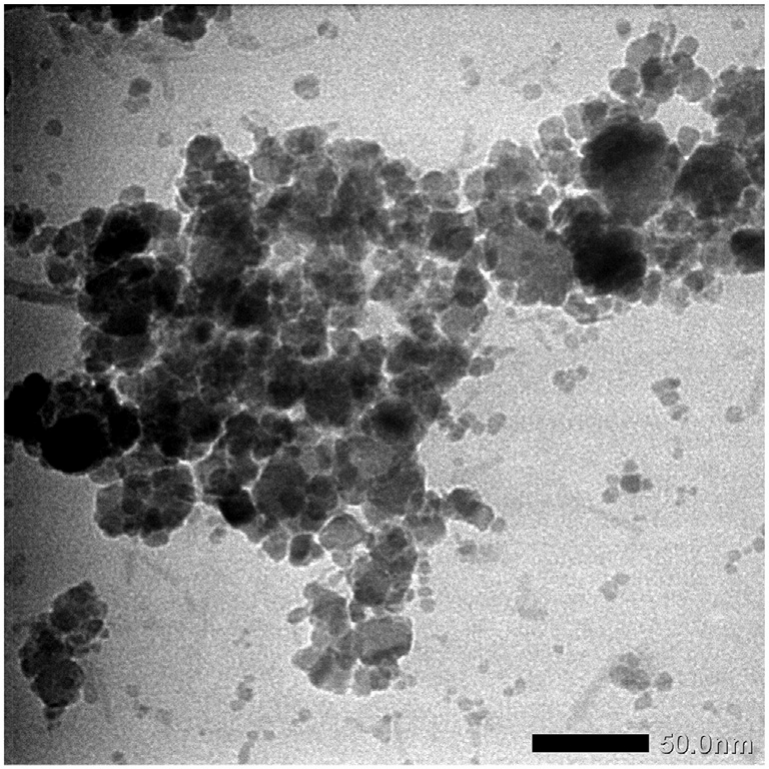
TEM image of SnO_2_–Fe_3_O_4_ nanocomposites.

SnO_2_–Fe_3_O_4_ nanocomposites were also characterized using TEM. The purpose of this was to determine the morphological properties of the SnO_2_–Fe_3_O_4_ nanocomposite by observing the crystal structure, identifying the defects, determining the particle size, analyzing the interface, and the microstructure. The results of the TEM analysis are shown in Figure 4.

Based on the TEM results above, the morphological or inner structure results showed that the nanocomposites had either aggregation or a unified structure. In Figure 4 the darker centre was the iron oxide nanoparticles and the bright part was the tin oxide, the results are in agreement with that of Nouri and Sargolzaei [21], where the darker dots appeared to spread out over the bright areas. Figure 4 shows the presence of a material that had a crystallographic field indicating SnO_2_. From this figure, it was also observed that there was an aggregate phase at the darker colour intensity at several points which showed the aggregation of Fe_3_O_4_ particles as shown in the TEM results of Wang et al. [22]. From the TEM results on Fe_3_O_4_, a large agglomeration and particles that tended to form clusters were discovered. This analysis was also supported by the condition of Fe_3_O_4_ nanoparticles which are prone to clumping due to their strong magnetic force. Based on the results of calculations by measuring the diameter at several particle points, the size of the SnO_2_–Fe_3_O_4_ nanocomposite averaged 6.5 nm indicating the size of a nanoparticle.

### 3.5 Characterization using SEM-EDS

**Figure 5:**
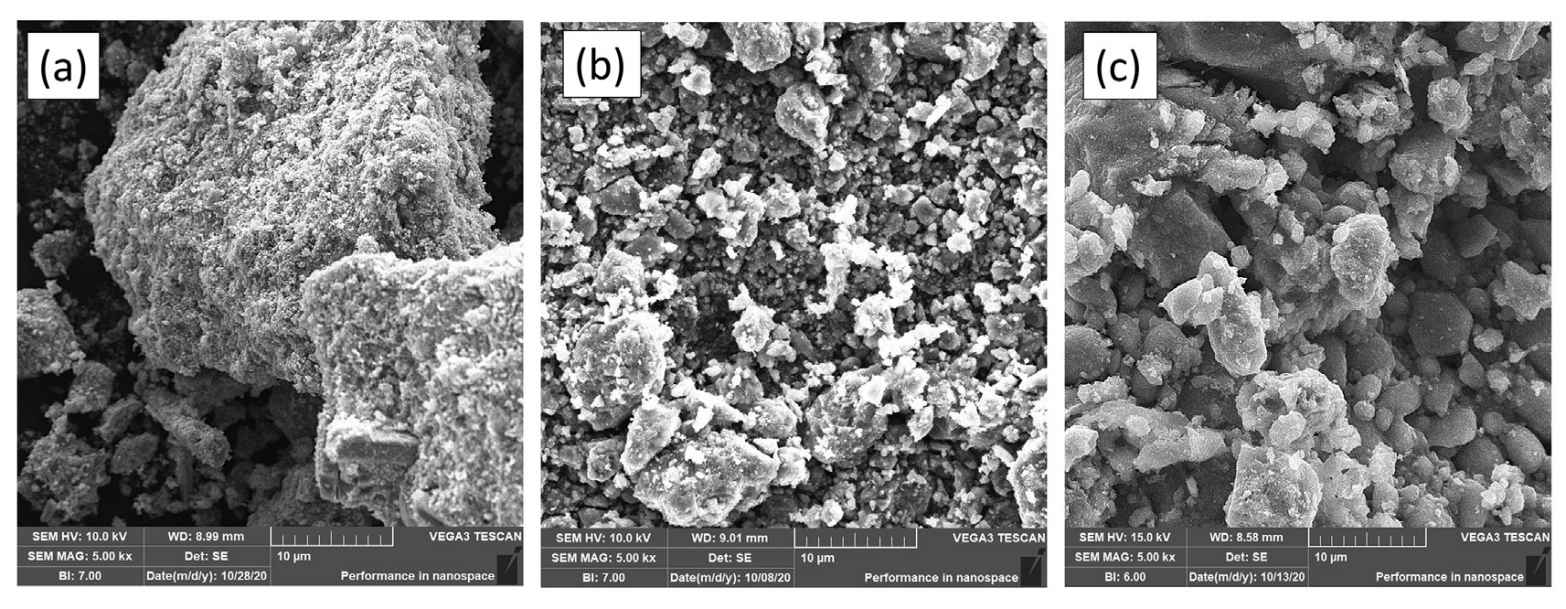
SEM morphology (a) SnO_2_, (b) Fe_3_O_4_ and (c) SnO_2_–Fe_3_O_4_ nanocomposites.

The surface of the SnO_2_–Fe_3_O_4_ nanocomposite was observed using SEM. As shown in Figure 5 (a). SnO_2_, Figure 5 (b). Fe_3_O_4_ and Figure 5 (c). SnO_2_–Fe_3_O_4_ nanocomposites.

Based on the SEM results in Figure 5 (a), the SnO_2_ structure was in the form of chunks, which had a non-smooth surface and formed agglomeration. Chu et al. [23] reported similar things in their research, where SEM images showed the results were in the form of aggregates and were composed of particles with a diameter of 10–20 nm Figure 5 (b) showed the surface of Fe_3_O_4_ which consisted of small, uneven particles and the presence of gaps in the pores. The Fe_3_O_4_ particles in the SEM results were not uniformly spherical and they formed agglomeration. This is because the particles have strong magnetic properties which tend to draw closer to one another. The morphology of the synthesized SnO_2_–Fe_3_O_4_ nanocomposite is represented in Figure 5 (c), showing that the gap between the pores was bigger than those in Figure 5 (b). This is due to the presence of SnO_2_ coating on the composite forming a core-shell structure. The results of Wang's study [24] showed that SnO_2_ coats and sticks to the outside of Fe_3_O_4_. The EDS results are observed in Table 2.

**Table 2:** EDS data on the composition of the constituent elements of SnO_2_, Fe_3_O_4_ and SnO_2_–Fe_3_O_4_ nanocomposites.

Sample	Element (% Mass)								Total (%)
	Fe	Sn	O	C	F	Co	Na	Si	
SnO_2_	-	72.49	21.39	4.73	1.39	-	-	-	100
Fe_3_O_4_	43.57	-	36.9	18.16	-	1.38	-	-	100
SnO_2_–Fe_3_O_4_ Nanocomposites	56.52	13.07	22.39	4.68	-	1.99	0.92	0.45	100

Based on the EDS results in Table 2 for SnO_2_, the main constituents were Sn and O, with the respective percentages of 72.49% and 21.39%. And the major constituents of Fe_3_O_4_ were Fe at 43.57% and O at 36.9%. In the SnO_2_–Fe_3_O_4_ nanocomposite, there was an increase in the percentage of Fe by 56.52% indicating that this metal is the major constituent of the nanocomposite, while the percentage of Sn element was 13.07%. This causes the pore gap size in the nanocomposite to become smaller. Based on the results of the EDS, impurities were also discovered in the form of F, C, Co, Na and Si. The presence of these impurities comes from the coating process and also during the nanocomposite washing process.

### 3.6 Characterization using FTIR

**Figure 6:**
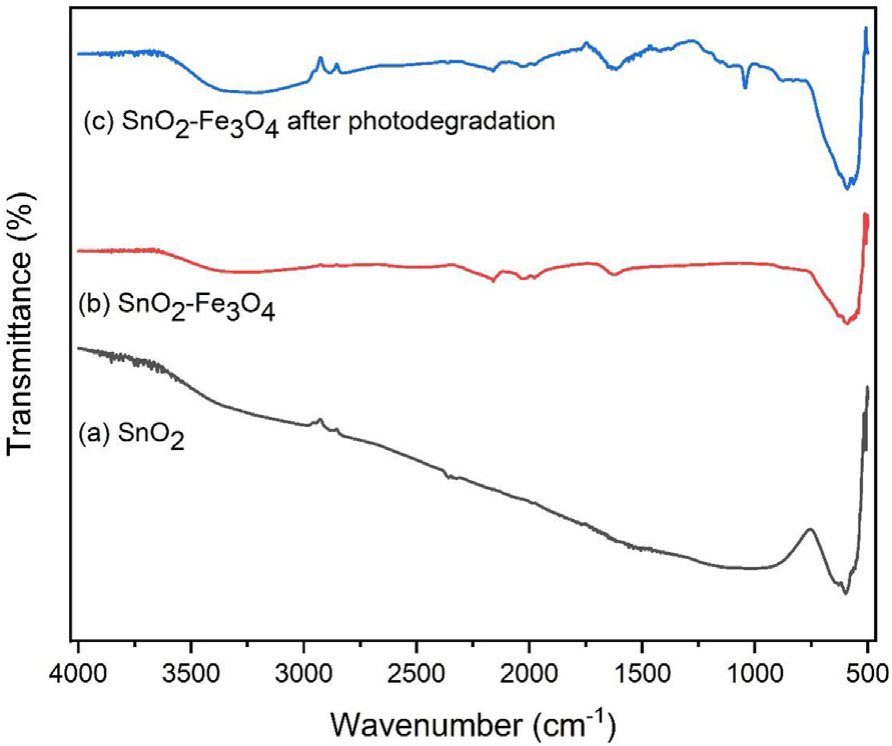
FTIR Spectrum (a) SnO_2_, (b) SnO_2_–Fe_3_O_4_ nanocomposites, (c) SnO_2_–Fe_3_O_4_ nanocomposites after photodegradation.

The synthesized SnO_2_–Fe_3_O_4_ and SnO_2_ nanocomposites were also characterized using FTIR. This was aimed to identify the functional groups present in the synthesized SnO_2_ and SnO_2_–Fe_3_O_4_ nanocomposites. The FTIR spectrum is presented in Figure 6.

Figure 6 showed that there was a significant change in the IR spectrum. In the FTIR spectrum of SnO_2_ nanocomposites shown in Figure 6 (a) an absorption band at 597 cm^−1^ was observed which was the peak characteristic of the Sn–O strain vibration on Sn–OH. While the FTIR spectrum of SnO_2_–Fe_3_O_4_ nanocomposites shown in Figure 6 (b), produced a wide absorption band that was observed at 3215 cm^−1^ on the O–H strain vibration. With the presence of an O–H absorption band that had a low transmittance, the crystal formation process was described as almost perfect. The absorbance band at 1626 cm^−1^ was described to represent the vibrations of the hydroxyl groups on the surface. The broad absorption peaks appearing between 400 cm^−1^ and 600 cm^−1^ were defined as the strain between metal and oxygen (M-O). The absorption band at 507 cm^−1^ was related to the vibration strain of Fe–O on Fe_3_O_4_ nanoparticles. Another absorption band at 590 cm^−1^ was characterized as the Sn–O strain band in SnO_2_ [25].

Figure 6 also shows that the changes and shifts were observed in the absorption bands within several spectra on the SnO_2_–Fe_3_O_4_ nanocomposites after photodegradation. In each spectrum, there was an OH group that appeared in the 3200-3600 cm^−1^ region. In Figure 6 (b) a water absorption vibration appeared on the O–H at 3212 cm^−1^, however in Figure 6 (c) the O–H vibration increased in the percent transmittance. At 590 cm^−1^, there was an increase in the transmittance of the nanocomposite before photodegradation (48.067%) and after (75.937%). At 563 cm^−1^, there was a change in the wave number of the nanocomposite after photodegradation to 507 cm^−1^ which indicated the effect of this process.

The results of characterization using XRD, UV-Vis DRS, VSM, TEM, SEM-EDS and FTIR showed that the SnO_2_–Fe_3_O_4_ nanocomposites were successfully synthesized by a combination of hydrothermal and coprecipitation methods. The SnO_2_–Fe_3_O_4_ nanocomposite had magnetic properties with a crystal size of 9.833 nm and an average particle size of 6.5 nm therefore, it was said to possess a nano size.

### 3.7 Point zero charge (PZC)

**Figure 7:**
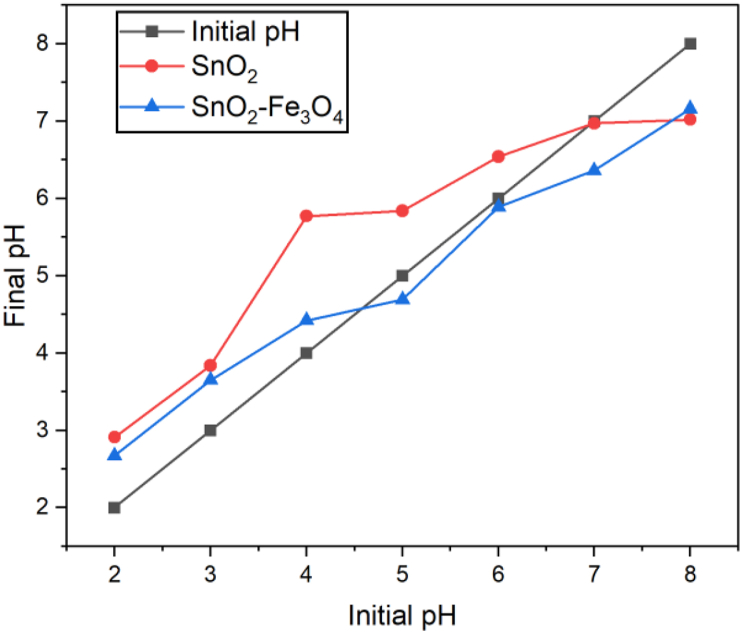
pHpzc measurement results.

SnO_2_–Fe_3_O_4_ and SnO_2_ nanocomposites were also analyzed by conducting a pHpzc determination test. The purpose of this was to ascertain the charge properties on the surface of the SnO_2_–Fe_3_O_4_ and SnO_2_ nanocomposites. pHpzc is a condition at a pH point, in which the surface of the nanocomposite has no charge i.e., its value is zero. This analysis uses pH drift, which is carried out by observing the shift in initial and final pH. The results of the pHpzc analysis are shown in Figure 7.

Based on Figure 7, the SnO_2_–Fe_3_O_4_ nanocomposite showed that there was no change between the initial pH value and that of the final at 6. SnO_2_ did not experience changes in the initial and final pH value at 7. According to Azeez et al. [26] the surface of the material is positively charged when the pH value is <pHpzc to allow the material to degrade an anionic compound. Meanwhile, the surface of the material becomes negatively charged when the pH value is > pHpzc, in order to degrade cationic compounds. In this study, the material used was applied for the photodegradation of the anionic congo red dye.

### 3.8 Determination of the optimum conditions for photodegradation of the Congo red dyes

#### 3.8.1 Variation in contact time and determination of kinetic parameters on the photodegradation of Congo red

**Figure 8:**
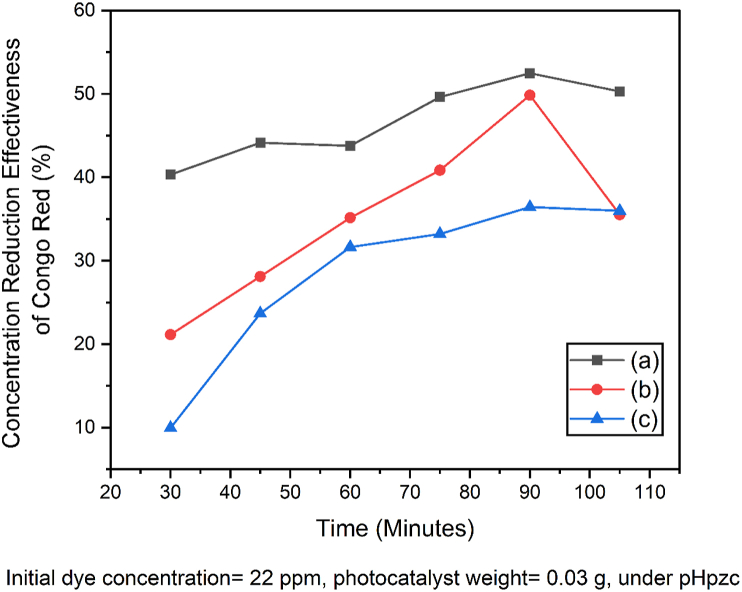
Effect of contact time for Congo red photodegradation by (a) SnO_2_–Fe_3_O_4_ Nanocomposites with UV irradiation, (b) SnO_2_ with UV irradiation (c) Nanocomposite SnO_2_–Fe_3_O_4_ without UV irradiation.

The variation of contact time was carried out to determine the optimum time for the photodegradation of the congo red dye by SnO_2_–Fe_3_O_4_ nanocomposites. The SnO_2_–Fe_3_O_4_ and SnO_2_ nanocomposites did not only act as photocatalysts, they also work as dye adsorbents during degradation. This is because both the SnO_2_–Fe_3_O_4_ nanocomposites and SnO_2_ have a wide surface and a porous structure [27]. Therefore, the photodegradation process of the SnO_2_–Fe_3_O_4_ nanocomposite without UV irradiation was carried out using the adsorption method. The following is the curve showing the effect of time on the amount of congo red dye as shown in Figure 8.

In Figure 8 the effectiveness of reducing the concentration of congo red dye with UV irradiation was observed to be higher than when it was without it. The percentage of reduction of the congo red dye with and without UV irradiation increased with time, this was because the longer the UV radiation time, the more photons or energy that hits the surface of the photocatalyst. The duration of irradiation at the time of photodegradation described the interaction time between the photocatalyst and UV light which produced free electrons (e^−^) and holes (h^+^) that later formed a redox reaction for the degradation of a compound. Nanocomposites have a limited ability to degrade a compound after reaching the optimum time. Based on the data obtained in Figure 8, the optimum time for photodegradation of congo red with and without UV irradiation was the same, i.e., at 90 min. At this optimum time, a balance had been reached to effectively reduce the dye concentration at 52.481%, 49.856% and 36.436%, respectively. This is because the ability of the photocatalyst to excite electrons from the valence band to that of the conduction did not increase and the resulting OH^•^ was widely used to degrade intermediates [28].

Based on the data obtained in Figure 8, it was observed that the effective reduction of congo red dye by SnO_2_–Fe_3_O_4_ nanocomposite with UV light irradiation was greater than when reducing the concentration of SnO_2_ using lamp irradiation and SnO_2_–Fe_3_O_4_ nanocomposite without UV light irradiation (control). Based on these data, it was stated that the presence of UV irradiation accelerated the decrease in the concentration of congo red dye.

The percentage decrease in the concentration of the congo red dye by the SnO_2_–Fe_3_O_4_ nanocomposite without UV irradiation was smaller than when it was with it. Therefore, it was stated that only adsorption occurred during photodegradation without UV irradiation. According to Paramarta [29], SnO_2_ is a metal oxide that shows its potential as an adsorbent. From the two statements, it was concluded that the photodegradation of the congo red dye by the SnO_2_–Fe_3_O_4_ nanocomposite using UV light irradiation, involved the adsorption process. Based on the time variable data, it was observed that the longer the photodegradation process, the higher the results.

Data on the effect of photodegradation time obtained was utilized to calculate the rate of degradation using the Pseudo-first-order and Pseudo-second-order model [30] as shown in Eqs. (1) and (2) as follow:

(1) $$\text{log ​}\left({\text{Q}}_{e}-{\text{Q}}_{t}\right)\;=\;\text{log}\;{\text{Q}}_{e}-\left(\frac{{\text{k}}_{1}}{2.303}\right)\text{t}$$

(2) $$\frac{1}{{\text{Q}}_{t}}\;=\;\frac{1}{{\text{K}}_{2}{\text{Q}}_{e}^{2}}\;+\;\frac{1}{{\text{Q}}_{e}}\;\text{t}$$

Where Q_e_ and Q_c_ were photodegradation capacity at equilibrium (mg/g), respectively, k_1_ (min^−1^) and k_2_ (g mg^−1^ min^−1^) were pseudo-first-order and second-order kinetic photodegradation rate constant, respectively, and t was a time (min).

The photodegradation rate is the ability of the SnO_2_–Fe_3_O_4_ and SnO_2_ nanocomposites to reduce congo red dye at a certain concentration and time. The kinetic model calculation data is presented in Table 3.

**Table 3:** Congo red dye kinetic model constant against the effect of time.

Treatment	Q_exp_ (mg/g)	Pseudo-first-order			Pseudo-second-order		
		Q_e_ (mg/g)	k_1_ (min^1^)	R^2^	Q_e_ (mg/g)	k_2_ (g.mg^1^. min^−1^)	R^2^
SnO_2_–Fe_3_O_4_ Nanocomposite with UV lamp irradiation	11.513	5.119	0.024	0.9418	12.422	0.006	0.9918
SnO_2_ with UV lamp irradiation	10.937	17.741	0.03	0.9725	24.27	0.003	0.9873
SnO_2_–Fe_3_O_4_ Nanocomposite Without UV lamp irradiation	7.993	23.93	0.048	0.9773	10.040	0.003	0.9830

In Table 3 Pseudo-second-order also showed that the value of equilibrium photodegradation capacity (Q_e_) in the experiment is not different from the value of Q_e_ obtained from the equation. Based on the data above, the photodegradation of SnO_2_–Fe_3_O_4_ nanocomposite with UV irradiation, SnO_2_ with UV irradiation, and SnO_2_–Fe_3_O_4_ nanocomposite without UV irradiation followed the Pseudo-second-order equation. The highest value of constant (k) was observed in the photodegradation process of SnO_2_–Fe_3_O_4_ nanocomposite with UV light irradiation. Knowing that the greater the value of the constant, the faster the reaction rate. A second-order kinetic model was also obtained by Nouri et al. [21] using SnO_2_–Fe_3_O_4_ nanocomposite for phenol photodegradation, while Vinosel et al. [25] utilized SnO_2_–Fe_3_O_4_ nanocomposite to degrade organic compounds following a first-order kinetic model. Based on the photodegradation kinetics, SnO_2_–Fe_3_O_4_ nanocomposite followed the pseudo-second-order equation.

#### 3.8.2 Variation of Congo Red's initial concentration of dyes

**Figure 9:**
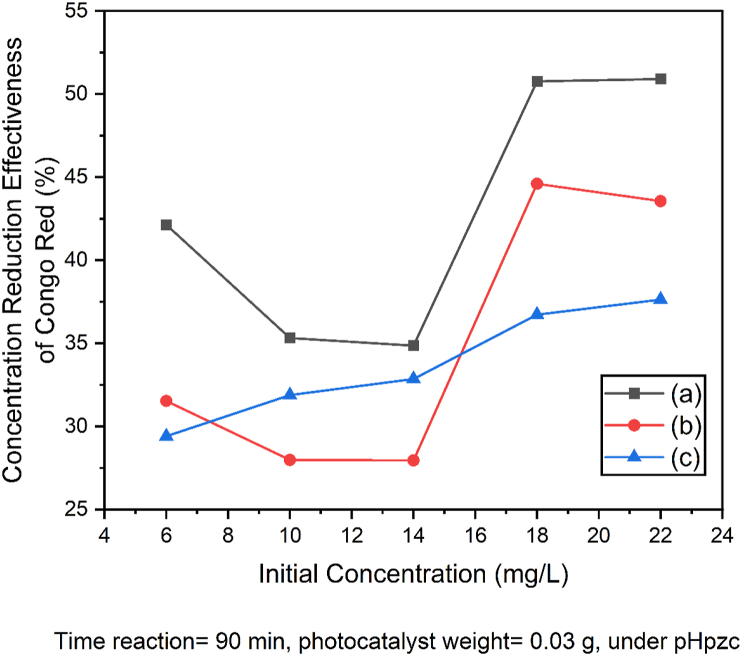
The curve of optimum photodegradation conditions with the effect of concentration (a) SnO_2_–Fe_3_O_4_ nanocomposite with UV irradiation (b) SnO_2_ with UV irradiation (c) SnO_2_–Fe_3_O_4_ nanocomposite without UV irradiation.

Variations in the initial concentration of congo red dye were carried out to determine the optimum concentration of photodegradation which occurred while utilizing SnO_2_–Fe_3_O_4_ nanocomposite. The curve for determining the optimum conditions for photodegradation of congo red dye on the effect of concentration is shown in Figure 9.

Figure 9 shows that the optimum concentration of photodegradation of Congo red dye with or without UV irradiation was 18 mg/L. At this condition, an equilibrium of concentration reduction was gradually reached at 50.768%, 44.600% and 36.723%. Based on Figure 9, a comparison was made between the percentage of concentration of Congo red dye that was reduced with and without UV irradiation. For the photodegradation process with UV irradiation, the concentration reduction was higher than without radiation. This is in accordance with Alshabanat's research [31] which stated that the UV lamp produced OH which helped to degrade Congo red dye. While treatment without UV irradiation only adsorbed the dye. Based on the results obtained, the concentration affected the percentage of photodegradation, I.e., the higher the concentration of congo red, the lower the percentage of photodegradation.

#### 3.8.3 Variation of volume addition of H_2_O_2_

**Figure 10:**
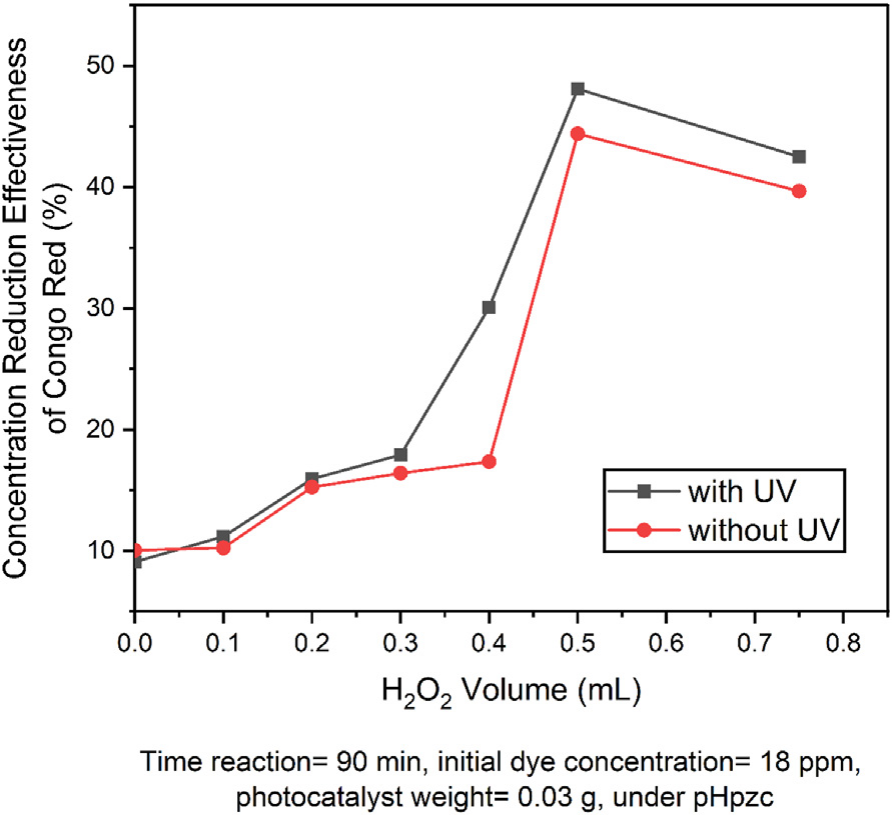
The curve of optimum conditions on the effect of adding the volume of H_2_O_2_ on photodegradation of congo red by SnO_2_–Fe_3_O_4_ nanocomposite.

Variations in the addition of H_2_O_2_ volume was aimed at determining the optimum volume of H_2_O_2_ that causes an increase in the reduction of the concentration of congo red. Congo red dye photodegradation effectiveness curve on the effect of adding H_2_O_2_ volume is observed in Figure 10.

This drastic increase was due to the higher H_2_O_2_ concentration that was added to the photodegradation of congo red by the SnO_2_–Fe_3_O_4_ nanocomposite. This led to an increased number of electrons in the conduction band which bounded to H_2_O_2,_ therefore preventing the occurrence of recombinant charges (e^−^) and (h^+^). OH continued to increase at the addition of 0.5 mL of H_2_O_2,_ while at 0.75 mL of H_2_O_2_, the percentage of effectiveness decreased because at that volume the less reactive HO_2_^•^ had started to form.

The optimum condition for the addition of H_2_O_2_ was 0.5 mL. According to Saha et al. [17], the effectiveness of the photodegradation decreased with the addition of an excess volume of H_2_O_2_ due to the formation of less reactive HO_2_^•^ which reacted with OH^•^, causing it to decrease. Based on the results obtained, it was observed that adding H_2_O_2_ to the photodegradation process while using SnO_2_–Fe_3_O_4_ nanocomposite had no effect.

### 3.9 Photodegradation mechanism

Photodegradation usually occurs on the semiconductor photocatalyst's surface after exposure to photons of light (UV). In the beginning, congo red adsorption on the surface of the nanocomposite had occurred through the electrostatic attraction between the sulfoxide (-SO_3_^–^) of the congo red molecule with the positive charge site of the nanocomposite [32]. Hereafter, photo-induced electron-hole pairs from nanocomposite can be produced during UV irradiation. When the photon energy is equal or higher to the bandgap of the semiconductor photocatalyst, electrons are promoted from the valence band to the conduction band, leaving an electron vacancy or hole in the valence band [33]. Since the energy level of Fe_3_O_4_ is lower than the conduction band of SnO_2_, photogenerated electrons will be transferred to Fe_3_O_4_, and this process is dynamically advantageous. Subsequently, electrons will react with oxygen molecules to produce reactive oxygen species (•O_2_^-^); as a result, the •O_2_^-^ can degrade congo red into CO_2_, H_2_O, NO_3_^-^, and NH_4_^+^ [34]. Photogenerated holes can react with OH ions to generate potent oxidizing hydroxyl radicals OH^•^, which can eminently degrade congo red as well [35]. In addition, the holes can also directly degrade when taken by organic compounds on the surface of the SnO_2_–Fe_3_O_4_ composite.

## 4 Conclusion

Based on the results of the study conducted, the following conclusions were obtained:

1. SnO_2_–Fe_3_O_4_ nanocomposite was successfully synthesized by the combined hydrothermal and coprecipitation methods. This was shown by the results of characterizations that were performed using XRD, UV-Vis DRS, VSM, TEM, SEM-EDS and FTIR.
2. Optimum photodegradation of congo red using SnO_2_–Fe_3_O_4_ nanocomposite was obtained at 90 min, when the concentration was 18 mg/L, and it produced a 50.76% degradation. Also, the results showed that the addition of H_2_O_2_ volume did not affect the photodegradation.
3. Congo red photodegradation kinetics by SnO_2_–Fe_3_O_4_ nanocomposite was according to the pseudo-second-order equation.

## Declarations

### Author contribution statement

Muhammad Said, Poedji Loekitowati Hariani: Conceived and designed the experiments; Contributed reagents, materials, analysis tools or data; Wrote the paper.

Widya Twiny Rizki: Performed the experiments; Analyzed and interpreted the data; Wrote the paper.

Wan Ryan Asri: Analyzed and interpreted the data; Wrote the paper.

Desnelli Desnelli: Performed the experiments; Contributed reagents, materials, analysis tools or data.

Addy Rachmat: Analyzed and interpreted the data; Contributed reagents, materials, analysis tools or data.

### Funding statement

This work was supported by Kementerian Pendidikan, Kebudayaan, Riset dan Teknologi Republik Indonesia (Penelitian World Class Research No. 150/E4.1/AK.04.PT/2021).

### Data availability statement

Data will be made available on request.

### Declaration of interests statement

The authors declare no conflict of interest.

### Additional information

No additional information is available for this paper.
